# Numerical study of critical straight, frown, and chevron incisions in small incision cataract surgery

**DOI:** 10.3389/fbioe.2023.1283293

**Published:** 2023-10-20

**Authors:** Yang Han, Nan Qi

**Affiliations:** ^1^ Research Center for Mathematics and Interdisciplinary Sciences, Shandong University, Qingdao, Shandong, China; ^2^ Institute of Marine Science and Technology, Shandong University, Qingdao, China; ^3^ Frontiers Science Center for Nonlinear Expectations, Shandong University, Qingdao, Shandong, China

**Keywords:** small incision cataract surgery, chevron incision, frown incision, energy release rate, finite element analysis

## Abstract

**Introduction:** When an intraocular lens (IOL) injector is inserted through a pre-cut corneal incision (e.g., an empirical size of 2.2 mm) during small incision cataract surgery, uncontrollable tearing to the corneal tissue may occur, which is highly associated with the incision shape, size, and location. The goal of this numerical study was to investigate the optimal incision scheme amongst three typical shapes, i.e., straight, frown, and chevron incisions using mechanical modeling and finite element analysis.

**Methods:** Assuming that the damage is caused by the tissue fracture at the incision tips and is governed by the classical energy release rate (ERR) theory which compares the current ERR value subject to IOL injection and the material’s intrinsic parameter, critical ERR *G*_*c*_.

**Results:** It was found that for chevron incisions, the incision shape with an angle of 170° was superior which induced minimal ERR value, while for frown incisions, the shape with a central angle of 6° was optimal. Both chevron and frown incisions could allow a larger size of injector to inject through than a straight pre-cut. In particular, the frown incision performed the best due to its lowest corresponding ERR and easy operation.

**Discussion:** It was also observed that regions where the embedded fibrils are more dispersed and exhibit high isotropy were more favorable. If necessary, the chevron incision was recommended to be more aligned with the direction exhibiting a larger modulus, for example, along the circumferential direction near the limbus. This study provides useful knowledge in operation design and a deep insight into mechanical damage to corneal wounds in small incision cataract surgery.

## 1 Introduction

Generally in routine cataract surgery, a key step is to insert a (manual or pre-loaded) intraocular lens (IOL) injector into an eye through a pre-cut incision. The goal of this surgery is not only to correct vision loss due to clouding of the lens, but also to create a watertight and stable incision to promote wound healing and reduce astigmatism Sinskey and Stoppel (1994). The length, shape, location of the incision, its relationship to the limbal, and its cross-sectional profile all contribute to wound healing and the amount of eventual astigmatism after surgery Shaikh et al. (2022), Sinskey and Stoppel (1994).

Over the past two decades, a small incision cataract surgery (SICS), a self-sealing cataract surgery has been widely reported. Due to the sclera-corneal tunnel construction, SICS has less postoperative response, faster and better visual recovery, fewer surgically induced astigmatism, and is easier to correct or control than conventional surgical methods Bernhisel and Pettey (2020), Al Mahmood et al. (2014), Kim et al. (2014). To allow safe insertion and secure incision integrity, this micro corneal incision (usually about 2.2 *mm* long) is carefully treated by surgeons to avoid irregular force-induced tissue damage Nanavaty and Kubrak-Kisza (2017), Kim et al. (2014), He et al. (2021), Zhang et al. (2022). Nanavaty and Kubrak-Kisza (2017) measured the length of the wound before and after IOL implantation to determine whether the wound was stretched.

**FIGURE 1:**
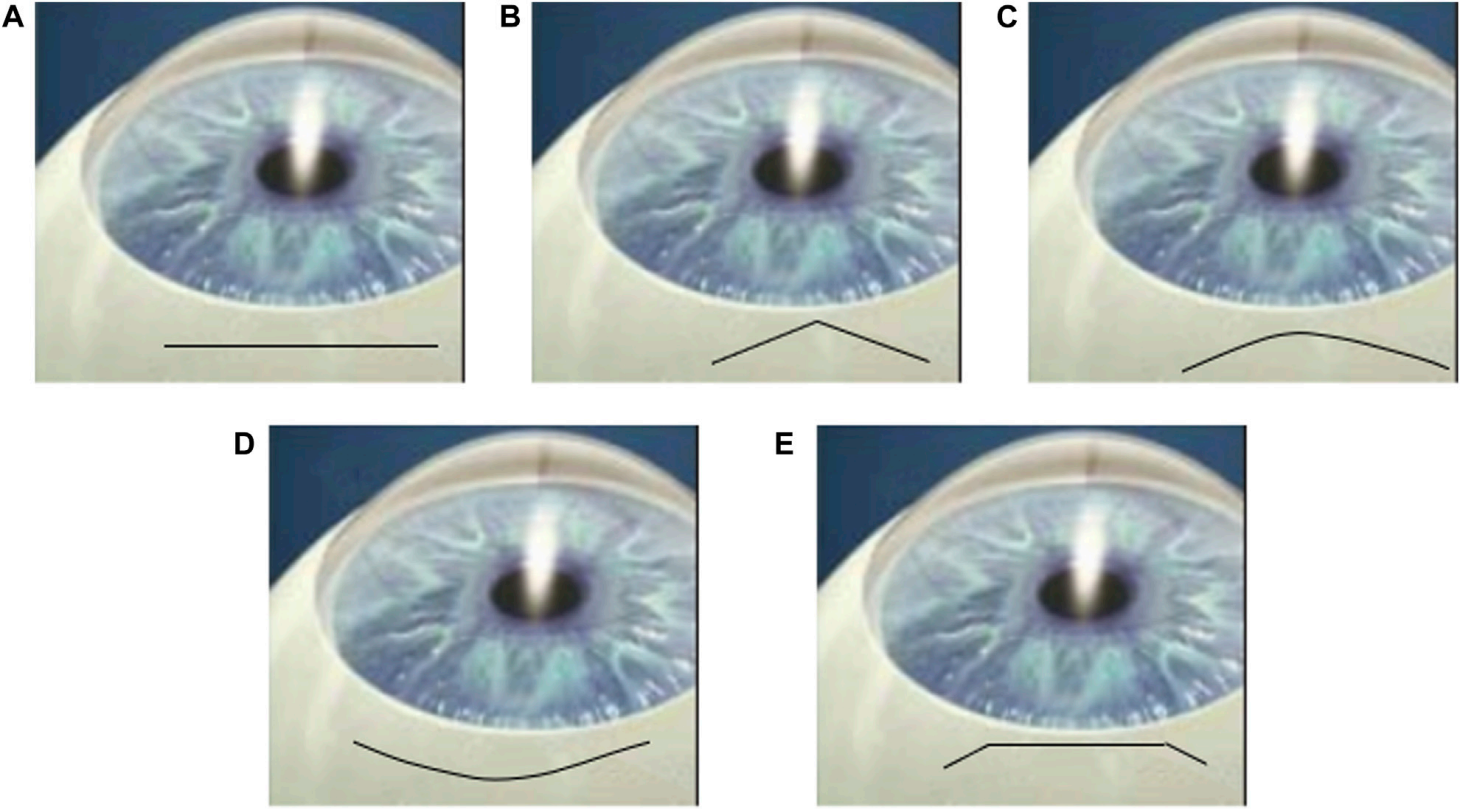
Cartoons of **(A)** straight, **(B)** chevron, **(C)** frown, **(D)** smile incisions and **(E)** Blumenthal side cut Haldipurkar et al. (2009).

The shapes of the incision in cataract surgery have attracted enough attention in clinical practice. McFarland first developed a sutureless straight incision in 1990 McFarland (1990), and Pallin described a chevron-shaped incision soon afterward (Pallin 1990; Pallin 1991). During the same period, Singer popularized the frown incision Singer (1991), which was later evidenced to be better than the straight incision in minimizing surgically induced astigmatism Chourasia et al. (2019). Other than these, there are many types of incision for tunnel construction such as smile incision and Blumenthal side cut Haldipurkar et al. (2009). The shapes of all these incisions are presented in Figure 1. Note that smile and frown incisions are both curved but facing the opposite.

In recent years, clinical and experimental studies have compared various incision shapes in the aspect of astigmatism Chourasia et al. (2019), Haldipurkar et al. (2009), Rathi et al. (2022). For example, the prospective study with a sample size of 100 patients concluded that a frown incision is evidently better than a straight incision in minimizing surgically induced astigmatism Chourasia et al. (2019). It was later pointed out that surgically induced astigmatism (SIA) through chevron incisions is the least followed by frown and straight incisions Rathi et al. (2022). However, the effects on wound integrity were scarcely explored and quantified, in particular from a mechanical viewpoint.

The work of Qi et al. (2022) focused on straight incisions in a cataract surgery. By constructing both analytical and numerical models to study the insertion of IOL during surgery, Qi et al. quantified the critical incision lengths according to the material energy release rate (ERR). Contributing factors such as insertion speed and injector size were also considered. However, all incisions were constrained as straight incisions. In this follow-on work, incision shape was the main focus, and a two-dimensional finite element model was constructed to simulate IOL insertion. Critical incision size to avoid wound tearing was quantified and optimal incision shape was selected amongst straight, chevron, and frown incisions.

## 2 Materials and methods

**FIGURE 2:**
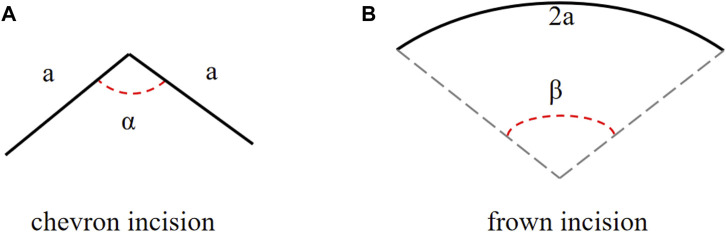
The schematic diagrams of **(A)** a chevron and **(B)** frown incisions, angles *α* and *β* are adjusted to represent different incision shapes.

**FIGURE 3:**
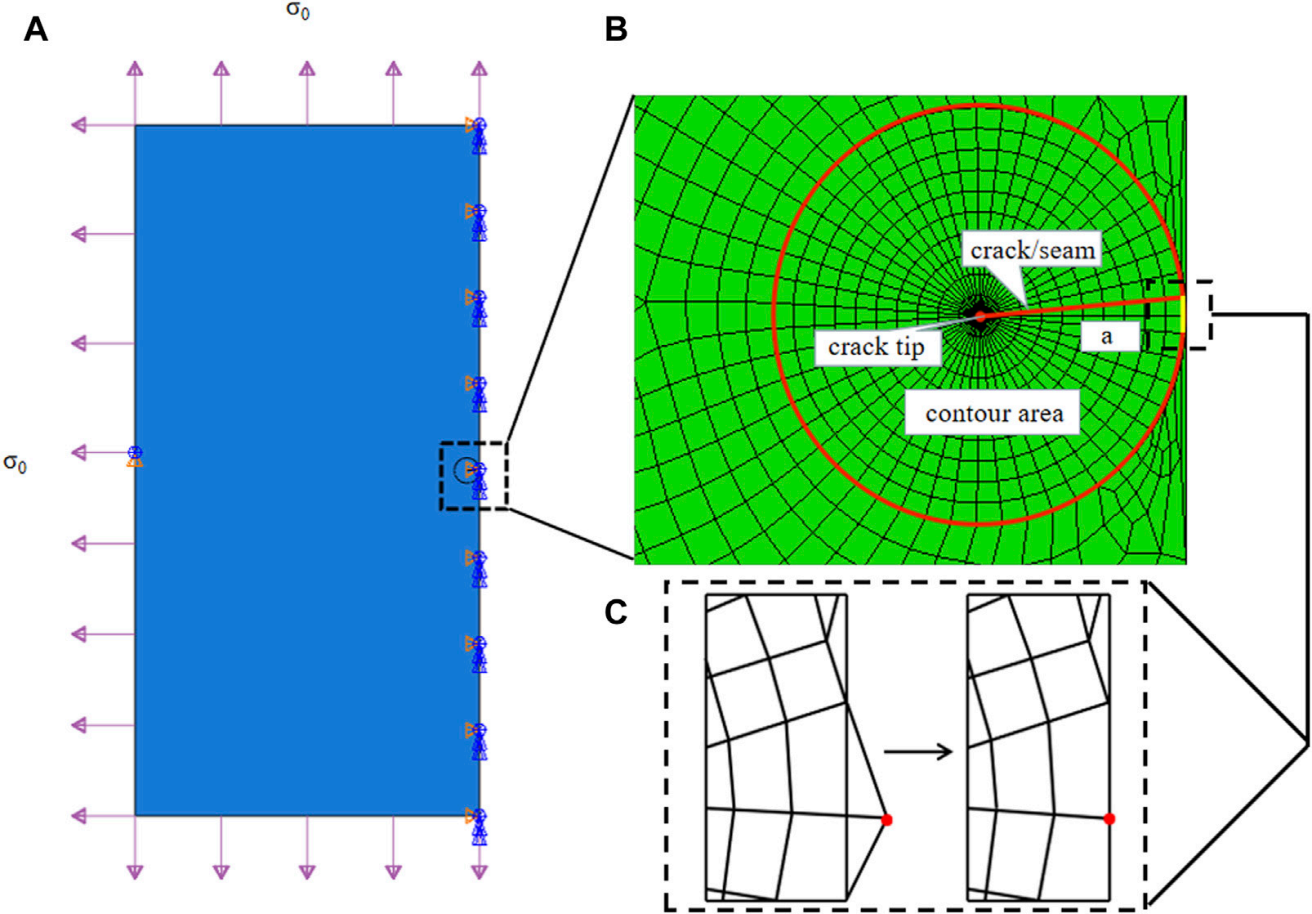
**(A)** Sketch of a two-dimensional plate torn by a typical chevron incision of length 2*a* in the middle under evenly distributed stress *σ*_0_ at each side. Note that in the finite element simulation, only half the problem was modeled. **(B)** The crack tip was surrounded by a spider web-like area for ERR calculation. The configuration of a chevron-shaped incision is defined in Figure 2A. **(C)** Due to the contour design, special treatment was required to pull nodes back onto the symmetrical axis, zooming in at the middle right corner of the designed FE mesh.

The finite element (FE) model was set up by considering a large two-dimensional square sample (60 *mm* by 60 *mm*) with a pre-existing chevron/frown incision (shapes shown in Figures 2A, B) in the middle with incision length 2*a* = 2.2 *mm*, as shown in Figure 3A. The sample size was chosen so that the incision size was relatively small in comparison to the cornea studied. Due to symmetry, only the left half of the model was simulated in the model. The classic mode I boundary condition was applied with a uniform distribution of stress *σ*_0_ at each edge. The central point on the left edge was fixed to avoid rigid body motion. Assuming that the diameter of the IOL tube is *d*, then the critical opening of the incision is required to be equal to *d* to guarantee smooth and safe insertion. In the simulation, we examined the IOL size of 1.73 *mm* averaged from six popular company products (Ultrasert, Eyecee, iSert, CT Lucia, iTec and Rayone, respectively) Nanavaty and Kubrak-Kisza (2017), Zhang et al. (2022), so that the amount of stress being applied was adjusted to induce a *d* = 1.73 *mm* maximal displacement in the middle of the incision.

The cornea was assumed as a simple linear material and its surface curvature was ignored. Typical strain-rate dependent stiffness *E* is chosen as 9.82 *MPa* at a strain rate of 3 *mm* ⋅ min^−1^ from porcine corneas Tonsomboon et al. (2014). In a chevron incision, the incision of length 2*a* was composed of two straight lines of the same length *a*, and the angle between these two arms is *α*, ranging from 120° to 180° Pallin (1991), Biswas et al. (2022) ^1^, as defined in Figure 2A. A frown incision was assumed to be an arc with arclength 2*a* and central angle *β*, as defined in Figure 2B. The central angle of a frown incision varied from 5° to 50° characterizing its curvature Sinskey and Stoppel (1994); Singer (1991) ^2^. As the corneal material is isotropic, a frown (curving down) and a smile incision (curving up) are the same.

In linear fracture mechanics, a 2D fracture occurs when the energy released by crack propagation along an infinitesimal length is greater than the energy required to break all atomic bonds per unit length, i.e., the crack becomes unstable. This idea is known as Griffith’s theory, the energy release rate criterion, or the G criterion Griffith (1921), Irwin and Wells (1965). The criterion is based on the calculation of energy release rate (ERR), *G*, which simply reads as

$$G>G_{c}.$$ (1)

where *G*_*c*_ is a measurable material parameter and its value at a strain rate of 3 *mm* ⋅ min^−1^ corresponds to stiffness of *E* = 9.82 *MPa* is 5.40 *kJ*/*m* ^2^ Tonsomboon et al. (2014). Thus, the key in FE simulation was to obtain the ERR value and compare it with *G*_*c*_, if *G* > *G*_*c*_, further tear may occur.

The crack was firstly represented by a partition and created by defining a “seam,” where nodes on elements on each side of the crack could be separated. ERR, as a contour energy integral, was calculated for layers of elements in rings, which required a spider web-like mesh generated around the crack tip. A sweeping strategy was selected around the crack tip, so that quadrilateral elements degraded into triangular elements, as seen in Figure 3B. In particular, each ring of elements along the crack corresponded to a contour integral ERR, and an average of 10 contours were selected for ERR convergence and evaluations. Note that for a chevron/frown incision, some special treatments were required to conduct effective sweeping operations and achieve a conforming and feasible mesh. One was to pull nodes back onto the symmetrical axis, these nodes escaped out of the plate due to the sweeping strategy, as depicted in Figure 3C, and the other was to remove coincident or nearly coincident nodes, especially for frown incisions.

The finite element simulations were performed using the commercially available finite element package Abaqus 2013 (Dassault Syst $\overset{`}{\text{e}}$ mes^®^), and was conducted on 11th Gen Intel^®^ Core^TM^ i5-11400@2.60GHz machine with 16.0 GB RAM. Abaqus script was modified to execute special sweeping operations for chevron/frown incisions via Matlab R2022a (The MathWorks, Inc.). In a typical simulation, a total of 17,882 nodes and 6,580 elements (CPS4R: Bilinear elements using simplified integration with hourglass control) were used, which took about 3–5 min. The grid size was chosen according to a grid independence test (simulations were run for increasingly refined grids until results converged).

## 3 Results

**FIGURE 4:**
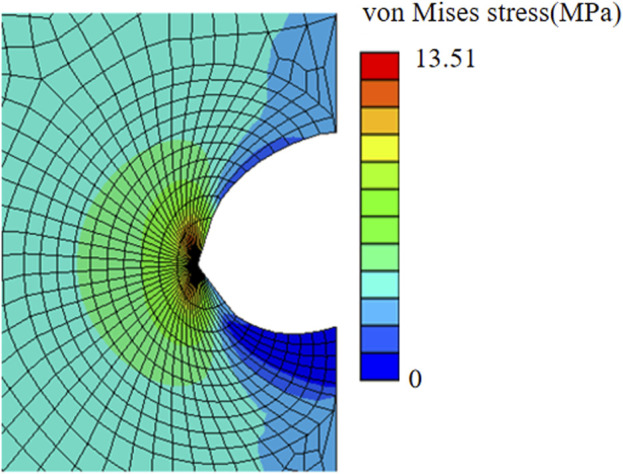
von Mises stress distribution of a chevron incision opening with incision length of 2*a* = 2.2 *mm* and angle between two arms of 150° subjected to the applied uniform stress of *σ*_0_ = 3.95 *MPa*.

Figure 4 shows a finite element result of a chevron incision with angle *α* = 150° and incision length of 2*a* = 2.2 *mm*, subjected to an applied loading of *σ*_0_ = 3.95 *MPa* to induce a maximal displacement of *d* = 1.73 *mm*. The crack opened unsymmetrically, more towards the chevron incision tip, and the von Mises stress was concentrated at the crack tip. The ERR corresponding to each contour of 10 typical contours was output from Abaqus. To avoid unnecessary effects from the crack tip, only contour integrals from the 4^th^ to 10^th^ contours were collected and all converged to a value of ERR of 5.12 *kJ*/*m* ^2^, which is less than *G*_*c*_ = 5.40 *kJ*/*m* ^2^ indicating safe insertion.

**FIGURE 5:**
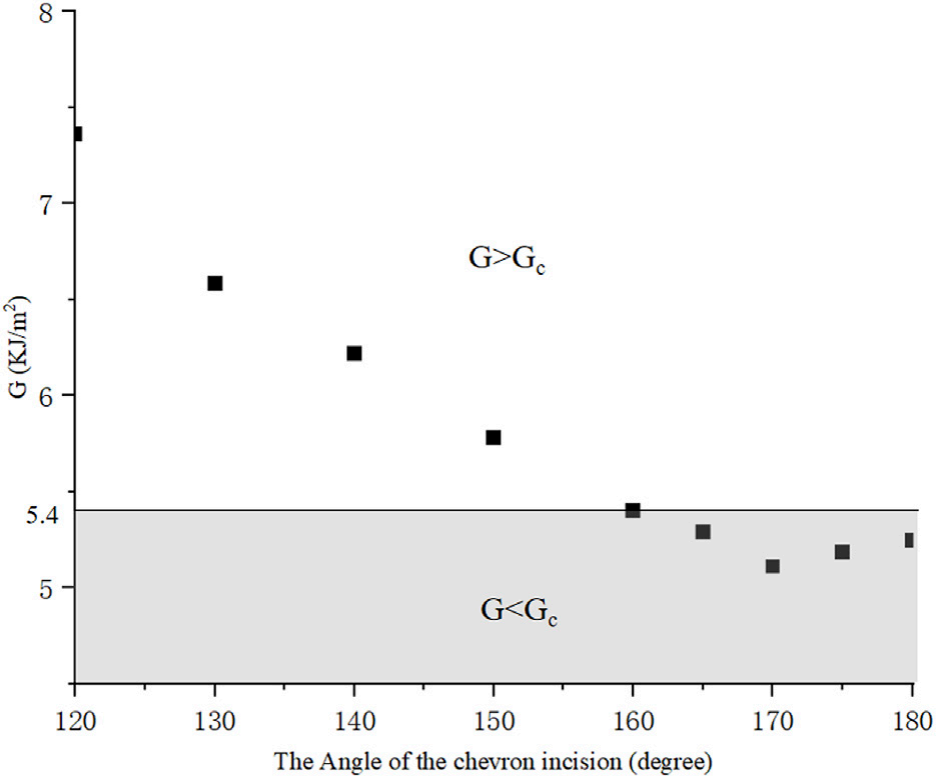
With different angles, ERR presents a tendency for chevron incisions with equal length 2*a* = 2.2 *mm*.

We then compared the ERR of chevron incisions with the angle *α* ranging from 120° to 180° and the same length of 2*a* = 2.2 *mm* Kim et al. (2014), as plotted in Figure 5. *G* equaled to the critical *G*_*c*_ = 5.40*KJ*/*m* ^2^ when *α* = 160°, and for *α* ≤ 160°, *G* ≥ *G*_*c*_, the crack further propagated, otherwise, remained stable. It was also found that *G* = 5.11*KJ*/*m* ^2^ reached the minimum value with *α* = 170°, which may indicate an optimal design for chevron-shaped incisions.

**FIGURE 6:**
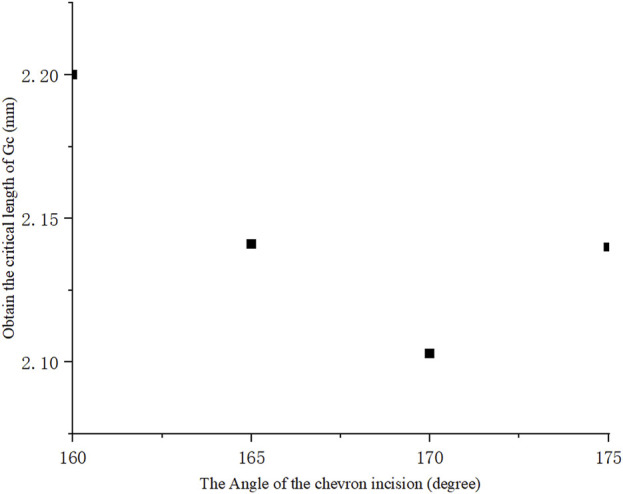
Critical incision length for different chevron shapes, the angle between two arms of 170° reached minimum indicating its optimal incision design.

To further investigate the critical length for different shapes of incision, four different chevron incisions with angles *α* = 160°, 165°, 170°, 175° were selected for numerical simulation, and their critical incision lengths corresponding to *G* = *G*_*c*_ were shown in Figure 6. It is worth noting that for each shape of chevron incision, the mesh needed to be regenerated as the angle between its two arms changed. It was clearly seen that the critical incision length is the smallest with *α* = 170°, i.e., with the least surgically induced damage, which is consistent with the observation that the ERR is minimum with this shape of chevron incision, as plotted in Figure 5. It is again convinced that the design of a chevron incision of angle 170° is optimal in order to maintain a stable corneal configuration.

**FIGURE 7:**
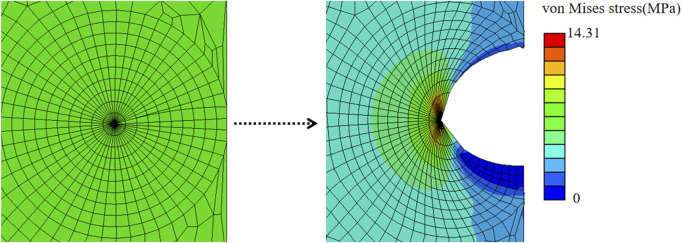
Simulation result of a frown incision with 2*a* = 2.2 *mm* and central angle of 35°, the incision is highlighted in red.

**FIGURE 8:**
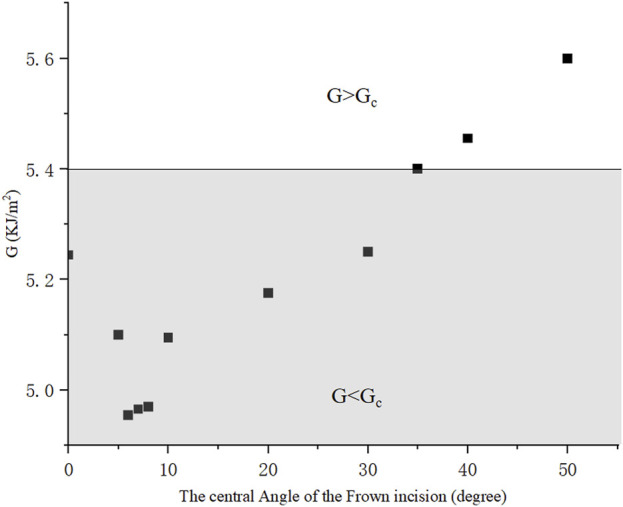
ERRs of frown incisions with different central angles, the incision length remained 2.2 *mm*.

Furthermore, frown incisions with the same length of 2*a* = 2.2 *mm* and different central angle *β* were simulated. A typical numerical example with *β* = 35° was elaborated in Figure 7. Similarly, by adjusting the evenly distributed stress at the boundaries, the value of ERR was taken when the maximum crack opening distance in the middle reached *d* = 1.730 *mm*. The crack opened asymmetrically more towards the convex direction and the maximum von Mises stress was concentrated at the crack tip. The contour integral ERR was extracted to converge to a value of 5.40*kJ*/*m* ^2^ from the 4^th^ to 10^th^ contours. Figure 8 then plotted the ERRs corresponding to different shapes of frown incisions with the central angle *β* ranging from 5° to 50°. It could be seen that the minimum ERR value of *G* = 4.95 *kJ*/*m* ^2^ was obtained when *β* = 6°. Within a small range of central angle between 5–8°, ERRs kept a small value. The frown design of *β* = 35° served as a threshold, for *β* ≤ 35°, then *G* ≤ *G*_*c*_, the 2.2 *mm* incision would not tear further; for *β* > 35°, the incision became unstable as *G* > *G*_*c*_. Note again that the mesh had to be regenerated for each shape of the frown incision as the central angle changed.

**FIGURE 9:**
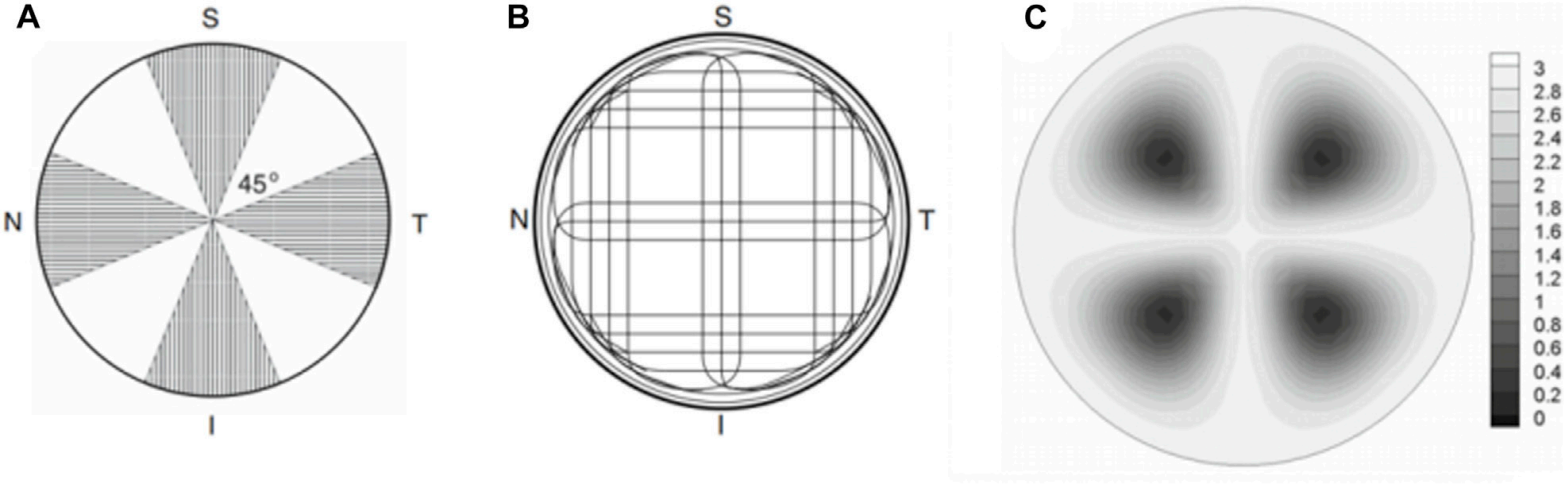
**(A)** Visualization of the corneal sector in which the strong direction of the fibrils is observed Pandolfi and Manganiello (2006); **(B)** Schematic diagram of the direction of human corneal fibers Meek and Newton (1999), Huang et al. (1996); **(C)** A graph of the coefficient defining the anisotropy level of fibrils. Small values correspond to isotropic distribution and large values correspond to significant anisotropy Pandolfi and Manganiello (2006).

This numerical model also allows us to incorporate more realistic corneal materials. It was well-known that the cornea has an anisotropy, and X-ray scattering measurements showed that about 66% of the fibrils were located in the 45° sector along the vertical and horizontal directions Pandolfi and Manganiello (2006), as seen in Figure 9A. In specific, the orientation diagram of human corneal fibrils is curved near the corneal limbus to form ring intensification and in the center of the cornea the fibril structure is positively intersected and runs around the rim of the cornea, showing a typical structure of a symmetrical material. as depicted inFigure 9B Meek and Newton (1999), Huang et al. (1996).

In order to study the influence of the material organization on ERRs, an orthotropic material whose moduli satisfied *E*_*x*_/*E*_*y*_ = *η* was applied, where *E*_*y*_ is the modulus parallel to the symmetric axis of the incision and *E*_*x*_ is the modulus perpendicular to *E*_*y*_, the othrotropic ratio *η* = 2, 1.5, 1, 0.75, 0.5, respectively. Table 1 lists the ERRS under different materials (*E*_*x*_, *E*_*y*_) and geometry (*α*). *G* is minimal at *η* = 1, indicating that the material is isotropic. The less the angle of *α*, the more effect of *η* on *G*.

**TABLE 1:** ERRs against the materials orthotropy ratio of chevron incision from different angles.

*η*	*E*_*x*_	*E*_*y*_	*G*			
			165°	170°	175°	180°
2	19.64	9.82	8.72	8.17	8.25	5.30
1.5	14.73	9.82	7.18	6.75	6.94	5.27
1	9.82	9.82	**5.29**	**5.11**	**5.19**	**5.24**
0.75	9.82	14.73	6.04	5.80	5.84	6.74
0.5	9.82	19.64	6.51	6.30	6.34	8.03

The bold values indicate the minimal ERR associated with each chevron angle at \eta = 1.

## 4 Discussion

In this work, we observed that with the same incision length of 2*a* = 2.2 *mm*, the frown incision shape with central angle *β* ≤ 20° exhibited at most 5.15% smaller ERR compared to a straight incision and thus better performance to maintain stable corneal configuration, which was consistent with the comment that the tendency for wound edge separation was comparatively less for frown structures Rao et al. (1993). It was also pointed out that frown incisions have the advantage of early wound stability over straight incisions Shaikh et al. (2022). Moreover, a chevron incision with an angle of 170° < *α* < 175° could achieve a smaller ERR than a straight incision with the same incision length, which was supported by a comparative study by Randeri et al. (2008), Rathi et al. (2022). However, the distinction was not big, only improved by 2.48%. Amongst the three shapes of incision, the frown incision performed significantly superior to the other two for allowing the largest injector through the pre-cut incision in an SICS. Other than this, a frown incision is practically easier to conduct for surgeons than a chevron incision which has a corner in the middle Singer (1991). It was found that the mean SIA value of the frown incision was lower than that of the straight incision, and the UCVA (uncorrected visual acuity) effect of the frown incision group was better than that of the straight incision group Chourasia et al. (2019). In summary, a frown incision is significantly better than a straight incision in reducing surgical astigmatism Chourasia et al. (2019).

The organization of corneal tissue is far more complex than being linear. It could be clearly seen from Table 1 that for chevron incisions, isotropy was favored among all tested angles, indicating practical incision locations were recommended in the dark regions in Figure 9C where the fibrils are most dispersed and exhibit high isotropy. Unlike straight incisions with *α* = 180°, *E*_*y*_ is no longer the dominating factor affecting ERR as the incision had both *x* and *y* components, the smaller the value of *α*, the more effect of *η* on *G*. If necessary, the chevron incision was recommended to be more aligned with the direction exhibiting a larger modulus, for example, along the circumferential direction near the limbus.

In further studies, finite element models with nonlinear and hyperelastic material descriptions are required based on patient-specific data. It is with no doubt that more complicated material descriptions would change the values in the results, however, the findings in this work are qualitatively representative of the clinical situations, and our simulation results are consistent with many comparative population studies and surgical findings. In addition, frown and smile incisions are required to be distinguished as curving to the rim and center represent differently in anisotropic material characterization. In the meanwhile, the cornea in this study was assumed to be a 2D plate, however, corneal curvature is an important geometrical parameter for accurate estimation, in particular, in cornea injury and its surgical repair Malik et al. (2012), Juchem et al. (1993), Merriam et al. (2003).

## 5 Conclusion

In this work, the finite element method was used based on Griffith’s energy release rate (ERR) criterion to simulate an optimal incision configuration among straight, frown, and chevron shapes, viewed from the point to maintain stable incision configuration and avoid further uncontrollable tearing in small incision cataract surgery. Chevron and frown incisions with different geometrical parameters including incision length and characteristic angles were considered and compared. It was found that for chevron incisions, the incision shape of angle 170° performed the best with minimal ERR value, while for frown incisions, the shape with a central angle of 6° was favorable. Both chevron and frown incisions could achieve larger sizes of injectors for surgeons to choose from than a straight pre-cut. In particular, frown incision was superior due to its lower corresponding ERR and easy operation. It was also observed that incision locations were recommended to be selected in regions where the embedded fibrils are most dispersed and exhibit high isotropy. If necessary, the chevron incision was recommended to be more aligned with the direction exhibiting a larger modulus, for example, along the circumferential direction near the limbus. This numerical study investigated and quantified the optimal incision shape in small incision cataract surgery from a new aspect, which provides a reference for ophthalmologists to make effective and safe surgical decisions.

## Data Availability

The original contributions presented in the study are included in the article/Supplementary Material, further inquiries can be directed to the corresponding author.

## Footnotes

1 Note that with *α* = 180°, a chevron incision coincides with a straight incision.

2 Note that with *β* = 0°, a frown incision coincides with a straight incision.
